# Supplementary figures and images for: Engineering T-Cell Resistance to HIV-1 Infection via Knock-In of Peptides from the Heptad Repeat 2 Domain of gp41
Source: mBio. 2022 Jan 25;13(1):e03589-21. doi: 10.1128/mbio.03589-21 (PMC8787484; doi:10.1128/mbio.03589-21)

**
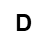

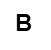

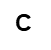

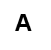

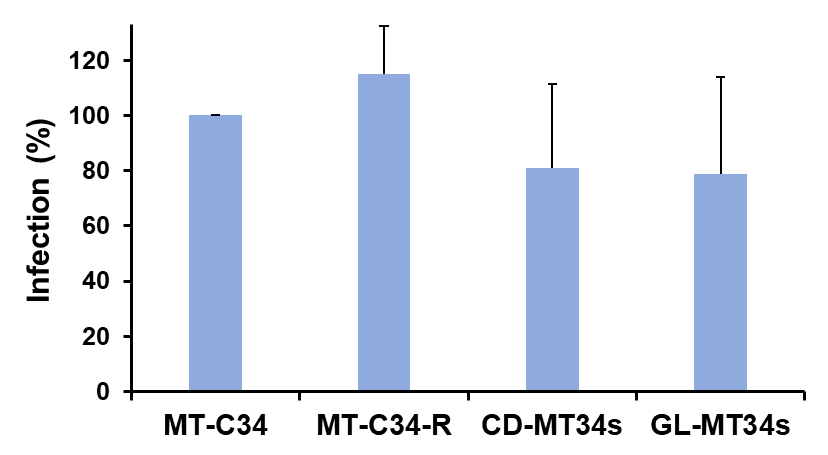

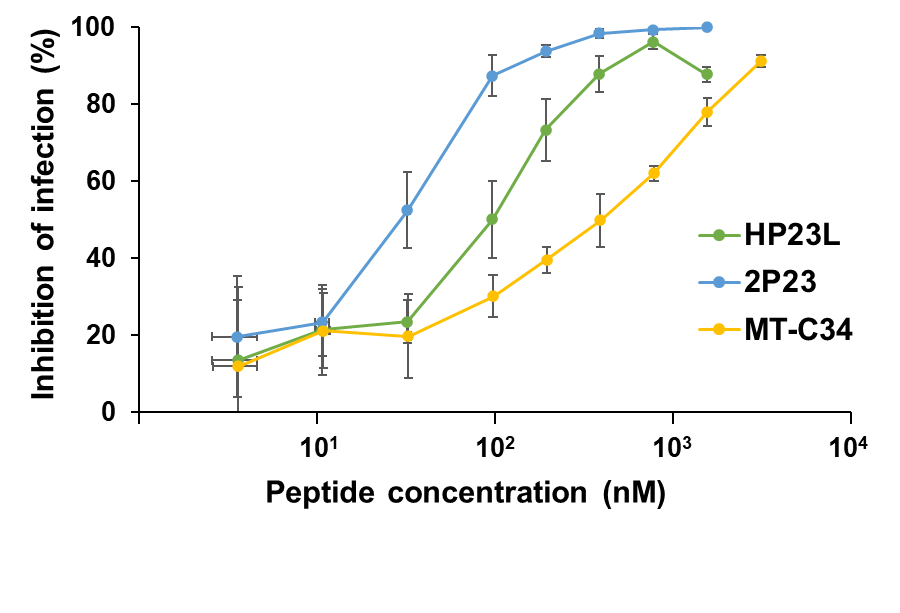

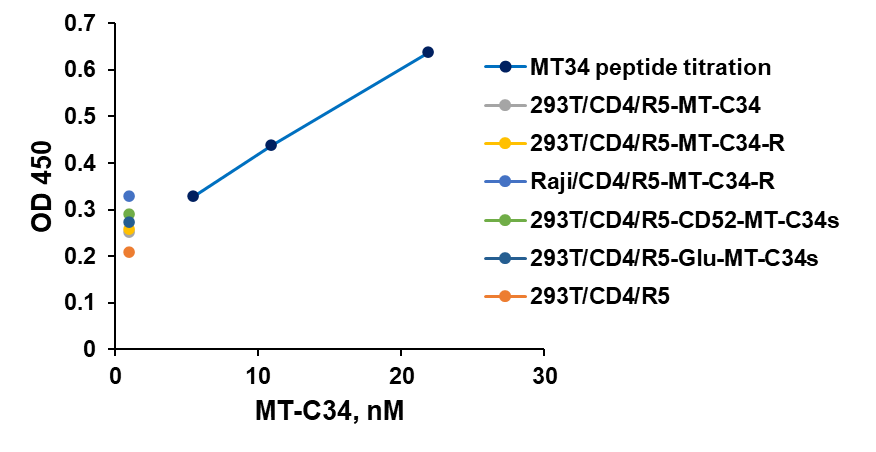

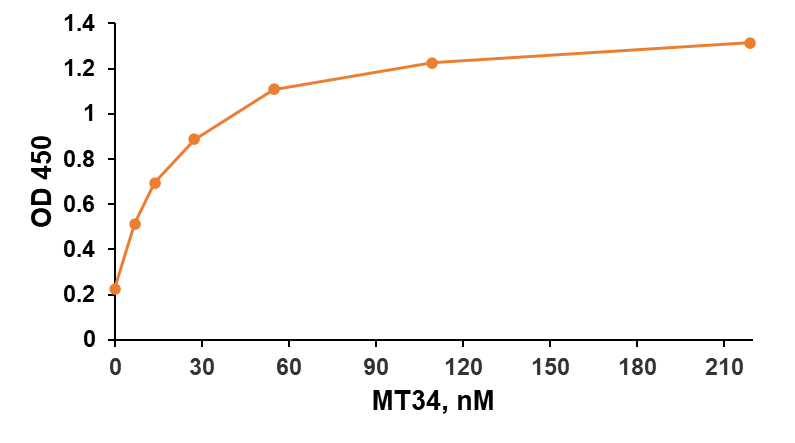
**

Supplement: FIG S1 [file mbio.03589-21-sf001.docx]

**
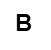

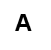
**

Supplement: FIG S3 [file mbio.03589-21-sf003.docx]

**
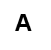
**

**
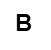
**

Supplement: FIG S4 [file mbio.03589-21-sf004.docx]
