# Supplementary material for: Engineering T-Cell Resistance to HIV-1 Infection via Knock-In of Peptides from the Heptad Repeat 2 Domain of gp41
Source: mBio. 2022 Jan 25;13(1):e03589-21. doi: 10.1128/mbio.03589-21 (PMC8787484; doi:10.1128/mbio.03589-21)
Supplement: TABLE S2 [file mbio.03589-21-st002.docx]

Table S2. Guide RNA and donor DNA generation.

| **Gene/**  **gRNA** | **Primers for donor generation** | **Templates** |
| --- | --- | --- |
| **CXCR4 start**  AGAACCAGCGGTTACCATGG***AGG*** | *5’­MT­C34(HA)­lp:͏͏* AGTTTGTTGGCTGCGGCAGCAGGTAGCAAAGTGACGCCGAGGGCCTGAGTGCTCCAGTAGCCACCGCATCTGGAGAACCAGCGGTT**ACCACCATGAAGCGCTTCC**  *3’­MT­C34(HA)­lp:͏͏* CGCTTCTGCCCGCTCGGAGAGGGGCTGCGCTCTAAGTTCAAACGTTTGTACATTTATGACAAAGCAGGTTGAAACTGGACTTACACTGATCCCCTC**GGCCGGTCCAGGATTCTC** | pUCHR-MT-C34-mClover or pUCHR­CD5HA2-mClover |
|  | *5’­MT­C34­F:͏͏* AGTTTGTTGGCTGCGGCAGCAGGTAGCAAAGTGACGCCGAGGGCCTGAGTGCTCCAGTAGCCACCGCATCTGGAGAACCAGCGGTTACC**ATGACCTGGATGGAGTG**  *3’­MT­C34­F:͏͏* CGCTTCTGCCCGCTCGGAGAGGGGCTGCGCTCTAAGTTCAAACGTTTGTACATTTATGACAAAGCAGGTTGAAACTGGACTTACACTGATCCCCTC**CAGGAGCTCCTGCTCG** | pUCHR-mClover-MT-C34 |
|  | *5’­C24­F:͏͏* AGTTTGTTGGCTGCGGCAGCAGGTAGCAAAGTGACGCCGAGGGCCTGAGTGCTCCAGTAGCCACCGCATCTGGAGAACCAGCGGTTACCATG**TACACCTCACTGATCCAC +** *3’­MT-C34­F* | pUCHR-mClover-MT-C34 |
|  | *5’­HA­F:͏͏* AGTTTGTTGGCTGCGGCAGCAGGTAGCAAAGTGACGCCGAGGGCCTGAGTGCTCCAGTAGCCACCGCATCTGGAGAACCAGCGGTTACCATG**TACCCTTACGACGTGCC**  *3’­HA­F:͏͏* CGCTTCTGCCCGCTCGGAGAGGGGCTGCGCTCTAAGTTCAAACGTTTGTACATTTATGACAAAGCAGGTTGAAACTGGACTTACACTGATCCCCTC**GGCGTAGTCGGGCAC** | pUCHR-mClover-CD5HA2 |
|  | *5’­2P23­F:͏͏* AGTTTGTTGGCTGCGGCAGCAGGTAGCAAAGTGACGCCGAGGGCCTGAGTGCTCCAGTAGCCACCGCATCTGGAGAACCAGCGGTTACCATG**GAGATGACCTGGGAAGAG**  *3’­2P23­F:͏͏* CGCTTCTGCCCGCTCGGAGAGGGGCTGCGCTCTAAGTTCAAACGTTTGTACATTTATGACAAAGCAGGTTGAAACTGGACTTACACTGATCCCCTC**CTTGAGCAGCTCTTCGATT** | pUCHR-mClover-2P23 |
|  | *5’­HP23L­F:͏͏* AGTTTGTTGGCTGCGGCAGCAGGTAGCAAAGTGACGCCGAGGGCCTGAGTGCTCCAGTAGCCACCGCATCTGGAGAACCAGCGGTTACCATG**GAGCTGACCTGGGAAGAG**  *3’­HP23L­F:͏͏* CGCTTCTGCCCGCTCGGAGAGGGGCTGCGCTCTAAGTTCAAACGTTTGTACATTTATGACAAAGCAGGTTGAAACTGGACTTACACTGATCCCCTC**CTTGAGGATCTCTTCGATTT** | pUCHR-mClover-HP23L |
| **CXCR4 exon-2**  cacttcagataactacaccg***agg*** | *5’­MT­C34­lp:͏͏* GCTTTAAAAATTTTTTTTAACTGGGTTAATGCTTGCTGAATTGGAAGTGAATGTCCATTCCTTTGCCTCTTTTGCAGATATACACTTCAGATAAC**GGATCCGGCGCAACAAAC**  *3’­MT­C34­lp:͏͏* GTAGATGGTGGGCAGGAAGATTTTATTGAAATTAGCATTTTCTTCACGGAAACAGGGTTCCTTCATGGAGTCATAGTCCCCTGAGCCCATTTCCT**CACACAAAAAACCAACACAC** | pUCHR-mClover-MT-C34 |
|  | *5’-X4-arm499bp-fwd:* TTTGCGCTTTAGGAGAATGAGT + *5’-X4-arm499bp-rev:* CCAATTCAGCAAGCATTAACCCA | Genomic DNA from PBMC |
|  | *3’-X4-arm440bp-fwd:* ACCCTGTTTCCGTGAAGAAAATG + *3’-X4-arm440bp-rev:* AGTCGGGAATAGTCAGCAGG |  |
| **CD4 start** AGGGACTCCCCGGTTCATTG***TGG*** | *5’­HA­lp:͏͏* GTTTGCTGACTAATGATTGGCATTTCCCTCAGGCCCTGCCATTTCTGTGGGCTCAGGTCCCTACTGGCTCAGGCCCCTGCCTCCCTCGGCAAGG**CCACCATGAAGCGCTTCC**  *3’­HA­lp:͏͏* GCCTTTCCTCCCACGTCATCTGCATTGAGACCCCAGGTCTGAGAACTTACCCAGTTGCAGCACCAGAAGCAAGTGCCTAAAAGGGACTCCCCGG**TTGGCCGGTCCAGGATTC** | pUCHR­CD5HA2-mClover |
| **CD4 stop-1**  gtagccccattTGAggcacg***agg***  **CD4 stop-2**  CTGGCCTCGTGCCTCAAATG***GGG*** | *5’­MT­C34(HA)­lp:͏͏* GGAGGTGCTAGAACGCAAAGGGGTTGCAGTGGGGACAGACCTGCTCCCCTTCTTCTTTGTTCCTGCAGCCGGTTTCAGAAGACATGTAGCCCCATT**GGATCCGGCGCAACAAAC**  *3’­MT­C34(HA)­lp:͏͏* GAGGCTGGGGATCTGCTACATTCATCTGGTCCGCAGGCAGGAAACGCGGGGCAGACACCTGGGGAGGCTGCAAGTGGGATCTGCCTGGCCTCGTGCC**TCAACTGAAGCAGAAGAGG** | pUCHR-mClover-MT-C34 or pUCHR-mClover-CD5HA2 |
|  | *5’-CD4-arm526bp-fwd:* CCTGGGCCATGTAACTGCTT + *5’-CD4-arm526bp-rev:* TGCAACCCCTTTGCGTTCTA | Genomic DNA from PBMC |
|  | *3’-CD4-arm536bp-fwd:* CAGATGAATGTAGCAGATCCCCA + *3’-CD4-arm536bp-rev:* ATGTGAGACATCCCTTGAGGC |  |
| **HIV-1 capsid**  GTTAAAAGAGACCATCAATG***agg*** | *5’­MT­C34­lp:͏͏* CCCATGTTTTCAGCATTATCAGAAGGAGCCACCCCACAAGATTTAAACACCATGCTAAACACAGTGGGGGGACATCAAGCAGCCATGCAAATGTTAAAA**GGATCCGGCGCAACAAAC**  *3’­MT­C34­lp:͏͏* CTAGTAGTTCCTGCTATGTCACTTCCCCTTGGTTCTCTCATCTGGCCTGGTGCAATAGGCCCTGCATGCACTGGATGCACTCTATCCCATTCTGCAGC**CACACAAAAAACCAACACAC** | pUCHR-mClover-MT-C34 |
|  | *5’-p24-arm530bp-fwd:* GTGCGAGAGCGTCAGTATTAA + *5’-p24-arm530bp-rev:* TTCTGATAATGCTGAAAACATGGG | pHIV-1-GFPt |
|  | *5’-p24-arm515bp-fwd:* GTGACATAGCAGGAACTACTAG  *+ 5’-p24-arm515bp-rev:* GCTATGTGCCCTTCTTTGCC |  |
| **Modification of donor plasmids with protospacers and DTS sequences** | | |
| **CXCR4 exon-2** | CACTTCAGATAACTACACCG***AGG*** cloned upstream of the donor at Not I/PspX I sites after annealing | pJet-X4ex2-don |
|  | ***CCT***CGGTGTAGTTATCTGAAGT cloned downstream of the donor at Xba I/Nco I sites after annealing |  |
| **HIV-1 p24** | ***CCT***CATTGATGGTCTCTTTTAAC cloned upstream of the donor at Xba I/Nco I sites after annealing | pJet-p24-don |
|  | GTTAAAAGAGACCATCAATG***AGG*** cloned downstream of the donor at Not I/PspX I sites after annealing |  |
| **DTS** | ATGCTTTGCATACTTCTGCCTGCTGGGGAGCCTGGGGACTTTCCACACCCTAACTGACACACATTCCACAG  cloned upstream of the p24 donor or downstream of X4ex2 donor at Xba I/Nco I sites after annealing | pJet-p24-don,  pJet-X4ex2-don |
| **Primers for targeted locus amplification** | | |
| **CXCR4 start** | 5’-gDNA: Cggactcactaccgaccac  3’-gDNA: gtacgggtacctccaatgtcc | Genomic DNA from CEM cells |
| **CXCR4 exon-2** | 5’-gDNA: GTATTTAGGCAGGCGTGGGA  3’-gDNA: AGTCATTGGGGTAGAAGCGG | Genomic DNA from CEM or CD4 lymphocytes |

Protospacer adjacent motifs are italicized and in bold. Primer sequences that have complementarity to the plasmid DNA template are shown in bold.
