# Supplementary material for: Engineering T-Cell Resistance to HIV-1 Infection via Knock-In of Peptides from the Heptad Repeat 2 Domain of gp41
Source: mBio. 2022 Jan 25;13(1):e03589-21. doi: 10.1128/mbio.03589-21 (PMC8787484; doi:10.1128/mbio.03589-21)
Supplement: FIG S2 [file mbio.03589-21-sf002.docx]

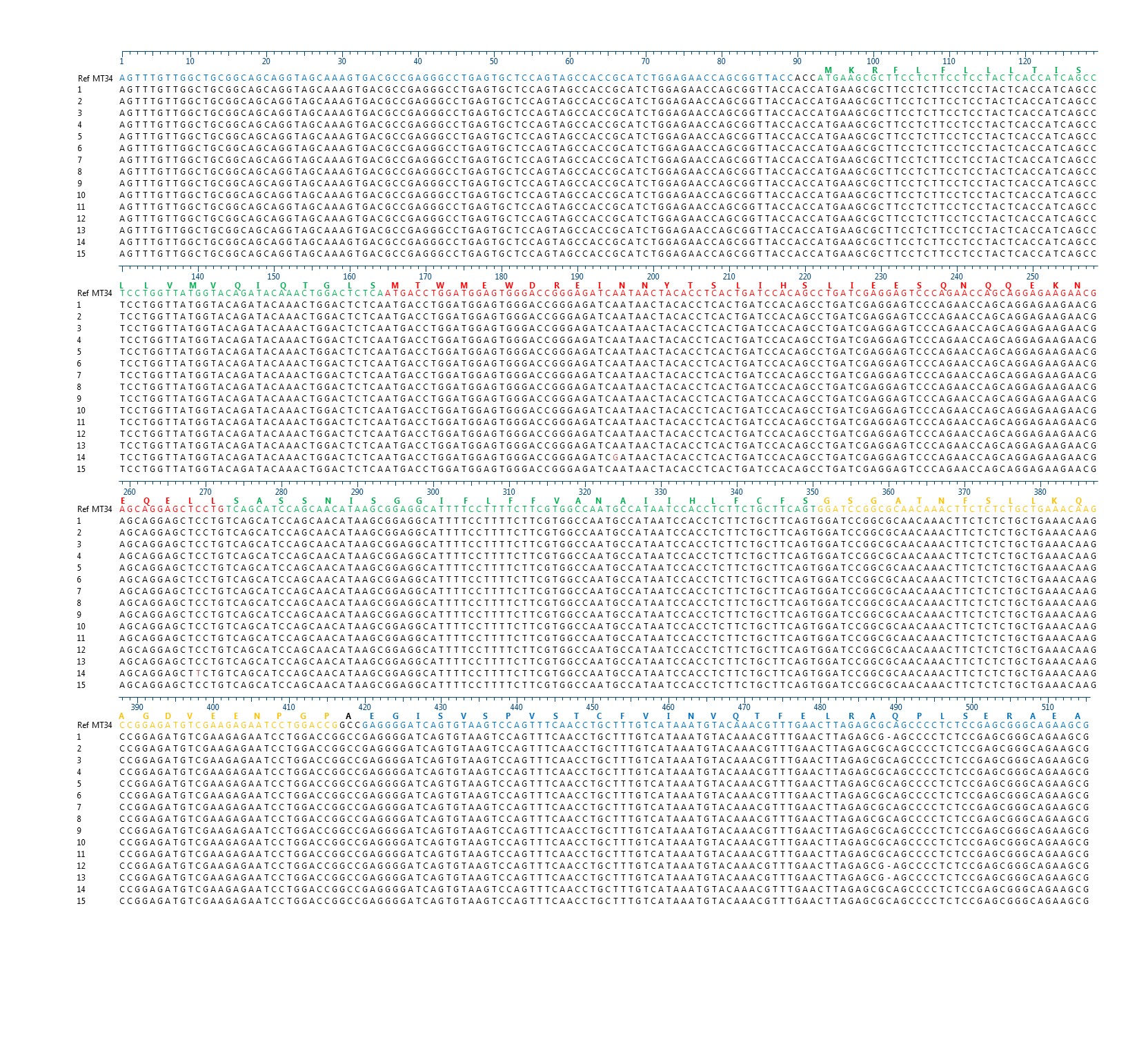


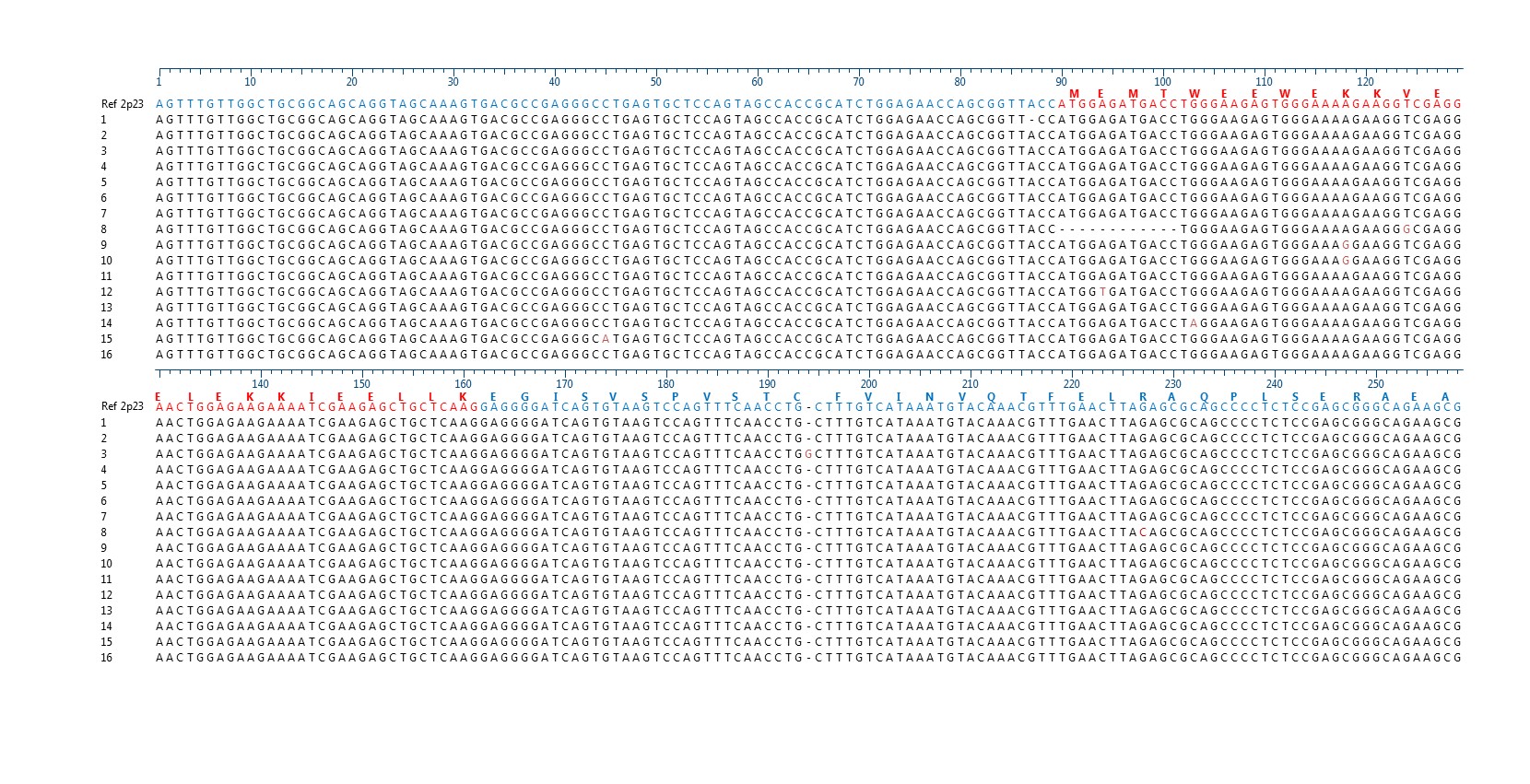


Homology arms and CXCR4 sequence

P2A sequence

Peptide sequences and mismatches

CD52 leader and GPI signals
