# Supplementary material for: Engineering T-Cell Resistance to HIV-1 Infection via Knock-In of Peptides from the Heptad Repeat 2 Domain of gp41
Source: mBio. 2022 Jan 25;13(1):e03589-21. doi: 10.1128/mbio.03589-21 (PMC8787484; doi:10.1128/mbio.03589-21)
Supplement: TABLE S1 [file mbio.03589-21-st001.docx]

Table S1. Primers used for plasmid construction.

| **Construct name** | **Primer name** | **Primer sequence** | **Templates** |
| --- | --- | --- | --- |
| **Generation of *pUCHR-mClover-CD5-peptide-2* constructs using fusion PCR and Afe I/PspX I restriction sites** | | | |
| Flanking primers | 5’-mClover | CTGTACAAGCCAGCCAAACCTCCGG | PCR products 1 + 2 |
|  | 3’-PspXI | CATTGGTCTTAAAGGTACTCGAG |  |
| C24 | 5’-C24  3’-C24 | GATCGAGGAGTCCCAGAACCAGCAGGAGAAGAACGAGCAGGAGCTCCTGTCAGCATCCAGCAACATAAG | pUCHR-mClover-CD5HA2 |
| C34 | 5’-C34  3’-C34 | TACACCTCACTGATCCACAG  CTGTGGATCAGTGAGGTGTAGTTATTGATCTCCCGGTCCCACTCCATCCAggtgtcgttttgtcctgag | pUCHR-mClover-C24 |
| MT-C34 | 5’-MT34  3’-MT34 | GgactctcaATGACCTGGATGGAGTGGGACCG  CCAGGTCATTGAGAGTCCAGTTTGTATCTG | pUCHR-mClover-C34 |
| HP23L | 5’-HP23L  3’-HP23L | GGAAAAGAAGATCGAGGAATACACCAAGAAAATCGAAGAGATCCTCAAGTCAGCATCCAGCAACATAAG  GTATTCCTCGATCTTCTTTTCCCACTCTTCCCAGGTCAGCTCTGAGAGTCCAGTTTGTATCTG | pUCHR-mClover-CD5HA2 |
| 2P23 | 5’-2P23  3’-2P23 | GAAAAGAAGGTCGAGGAACTGGAGAAGAAAATCGAAGAGCTGCTCAAGTCAGCATCCAGCAACATAAG  CAGTTCCTCGACCTTCTTTTCCCACTCTTCCCAGGTCATCTCTGAGAGTCCAGTTTGTATCTG | pUCHR-mClover-CD5HA2 |
| MT-WQ-IDL | 5’-WQ-IDL  3’-WQ-IDL | CACCAAAAAGATCGAAGAGCTCATCAAGAAATCCCAGAACCAGCAGATCGACCTGTCAGCATCCAGCAACATAAG  GCTCTTCGATCTTTTTGGTGTATTCCTCGATCTTCTTGTCCCACTCTTCCCAGGTCATTGAGAGTCCAGTTTGTATCTG | pUCHR-mClover-CD5HA2 |
| P52 | 5’-P52  3’-P52 | CCGAAGAGCAGCAGAAAAAGAACGAGGAAGAGCTGAAGAAGCTGGAGAAGTCAGCATCCAGCAACATAAG  CTTTTTCTGCTGCTCTTCGGCTTTCTTGAGCAGTTCCTCGATCTTCTGCTCCCATGAGAGTCCAGTTTGTATCTG | pUCHR-mClover-CD5HA2 |
| MT-C34-R | 5’-34furin  3’-34furin | CTGCGGATCAGGCGCTCAGCATCCAGCAACATAAG  GAGCGCCTGATCCGCAGGAGCTCCTGCTCG | pUCHR-mClover-MT-C34 |
| MT-C34-15D | 5’-34-15D  3’-34-15D | CAACATCAGCAGGGCCGACTGGAACGACGGCTCAGCATCCAGCAACATAAG  CGGCCCTGCTGATGTTGCAGTGGGCCTTCCTGATCAGGAGCTCCTGCTCG | pUCHR-mClover-MT-C34 |
| CD52-MT-C34s^*^ | 5’-mClover  3’-MT34-tr | CTGTACAAGCCAGCCAAACCTCCGG  GTACTCGAGCTCACAGGAGCTCCTGCTCG | pUCHR-mClover-MT-C34 |
| Gl-MT-C34s^*^ | 5’-Gl-LD  3’-MT34-tr | AAGCGCTTCGTTCTGTTTGCCCTGATCTGCATCGCTGTGGCCGAGGCTATGACCTGGATGGAGTGG  GTACTCGAGCTCACAGGAGCTCCTGCTCG | pUCHR-mClover-MT-C34 |
| ^*^ constructs were generated by a standard PCR with two primers | | | |
| **Generation of *pUCHR-MT-C34-mClover* construct using fusion PCR and Afe I/Age I restriction sites** | | | |
| Flanking primers | 5’-Afe I | GAAGCGCTTCCTCTTCCTC | PCR products 1 + 2 |
|  | 3’-Age I | CTCACCGGTGGCCATCC |  |
| N-MT-C34 | 5’-52-P2A | CCACCTCTTCTGCTTCAG | pUCHR-CD5HA2-sAID-mClover |
|  | 3’-52-P2A | CTGAAGCAGAAGAGGTGG | pUCHR-mClover-MT-C34 |
| **Construction of *pUCHR-CCR5* plasmid using EcoR I/Xma I restriction sites** | | | |
| CCR5 | 5’-EcoR-R5 | cGAATTCgccaccATGGATTATCAAGTGTCAAGTCC | cDNA from U937 cells |
|  | 3’-Xma-R5 | gCCCGGGTCACAAGCCCACAGATATTTCC |  |
| **Generation of *pNL4-3* with mutations in gp41 using overlapping primers and Bsa BI/Bam HI sites of restriction** | | | |
| Flanking primers | 5’-NB-BsaBI | CCATCAGTGGACAAATTAGATG | PCR products 1 + 2 |
|  | 3’-BamH-NB | GATCGTCCCAGATAAGTGC |  |
| V38A | 5’-V38A | CTGGTATAgcgCAGCAGCAGAACAATTTGC^*^ | pNL4-3 |
|  | 3’-V38A | GCTGCTGcgcTATACCAGACAATAATTGTCTGGCC |  |
| E49K | 5’-E49K | GAGGGCTATTaagGCGCAACAGCATCTGTTG |  |
|  | 3’-E49K | GTTGCGCcttAATAGCCCTCAGCAAATTGTTC |  |
| N126K | 5’-N126K | GAGAAATTAACaagTACACAAGCTTAATACACTCCTT |  |
|  | 3’-N126K | GCTTGTGTActtGTTAATTTCTCTGTCCCACTC |  |
| E136G | 5’-E136G | CACTCCTTAATTggaGAATCGCAAAACCAGCAAGA |  |
|  | 3’-E136G | CGATTCtccAATTAAGGAGTGTATTAAGCTTG |  |
| * The triplets coding mutated amino acids are shown by lowercase, and mutation is highlighted in red | | | |
